# A Novel Combination Therapy Approach Targeting STAT3 and Autophagy in Glioblastoma

**DOI:** 10.1080/27694127.2022.2117340

**Published:** 2022-09-06

**Authors:** Sujoy Bhattacharya, Lawrence M. Pfeffer, Edward Chaum

**Affiliations:** a Department of Ophthalmology and Visual Sciences, Vanderbilt University Medical Center, Nashville, TN; b Department of Pathology and Laboratory Medicine, The Center for Cancer Research, College of Medicine, University of Tennessee Health Science Center, Memphis, TN

**Keywords:** AMPKα, apoptosis, caspase 3, cathepsin D, LC3-I/LC3-II, MTORC1, RAD001 (everolimus), ULK1

## Abstract

The aggressive brain cancer glioblastoma (GBM) is notoriously resistant to radiotherapy and chemotherapy, which drives tumor recurrence and relapse. GBM cells are highly addicted to STAT3 (signal transducer and activator of transcription 3) and STAT3 inhibition blocks GBM-driven tumor growth. STAT3 regulates macroautophagy/autophagy, a central player in GBM pathobiology. Although autophagy suppression has been implicated in the pathophysiology of GBM, it is unknown if modulation of autophagy can reduce GBM tumorigenesis. Based on observations from our recent study, the answer appears to be yes, and we propose a therapeutic strategy for dual inhibition of STAT3 and MTOR, or STAT3 and ULK1 to target GBM tumorigenesis and chemoresistance.

Glioblastoma is the most common malignant brain tumor with a 5-year survival rate of ~5%. GBM is resistant to conventional chemotherapeutic drugs. There is a gap of knowledge regarding the mechanisms involved in GBM tumor recurrence and markers that can identify the cellular processes responsible for GBM tumor heterogeneity and chemoresistance. Understanding the molecular mechanisms that regulate GBM heterogeneity is required to develop new strategies to treat GBM.

The STAT3 signaling pathway drives malignant transformation of cells and STAT3 activation has been observed in a variety of tumor cells. STAT3 is constitutively phosphorylated in GBM and inhibition of STAT3 strongly attenuates GBM-driven tumor growth showing that GBM cells are addicted to STAT3. Analysis of brain tumors detected STAT3 overexpression in more than half of the patients with grade IV GBM cells, validating STAT3 as an important clinical target in GBM therapy. Supporting this, our studies demonstrate STAT3 post-translational modifications (PTMs) play a critical role in GBM tumorigenesis and its therapeutic resistance.

Recent studies have implicated STAT3 in the regulation of autophagy, a central cellular process linked to tumorigenesis. Both STAT3 signaling and autophagy have been investigated in glioma biology, but the role of autophagy in GBM tumor initiation and progression is still controversial, given its dual regulatory function in both cell death and survival. While STAT3 activation correlates with inhibition of autophagy, conflicting studies have also shown that STAT3 positively regulates autophagy. These differences may be explained by various STAT3 PTMs and altered Intracellular localization of STAT3. The autophagy activator, sirolimus ABI-009 (an albumin-bound MTOR kinase inhibitor) induces tumor regression in GBM and is currently being used in clinical trials for GBM patients. Although rapalogs such as everolimus have failed to demonstrate therapeutic efficacy, the idea of targeting MTOR in GBM with improved inhibitors has been resurrected. The novel compound PP242 targets both MTORC1 and MTORC2, and inhibits cell proliferation, stemness, and invasiveness in various GBM lines. Furthermore, histone deacetylase (HDAC) inhibitors vorinostat and tinostamustine are also emerging as potent therapeutic agents for the treatment of GBM possibly through modulating autophagy. The use of HDAC inhibitors in combination with MTOR and/or STAT3 inhibitors may represent an effective strategy to enhance GBM chemosensitivity by modulating autophagy.

To understand the relationship between STAT3 activation and autophagy in GBM cells, we used CRISPR-Cas9 knockout (KO) of *STAT3* in GBM cells and found that *STAT3*-KO activates autophagy, as shown by SQSTM1/p62 degradation and CTSD (cathepsin D)-associated lysosomal activity [1]. Treatment of *STAT3*-KO GBM cells with bafilomycin A_1_ increases LC3-II and SQSTM1 puncta formation, and reconstitution with wild-type (WT)-STAT3 in *STAT3*-KO cells completely reverses these effects, demonstrating that STAT3 activation suppresses autophagy. Similarly, phosphorylation defective Y705F and S727A STAT3 mutants also increase autophagy, providing evidence for the role of these phosphorylation events in suppressing autophagy in GBM cells. In addition, we found that *STAT3*-KO activates PRKAA/AMPKα-ULK1 signaling without affecting MTOR activation. This finding suggested that autophagy induction in these cells depends upon PRKAA/AMPKα-ULK1 but is MTOR independent. However, the catalytic MTOR inhibitor everolimus (RAD001) potentiates autophagy flux by inhibiting basal MTOR activity in *STAT3*-KO cells and cells expressing phosphorylation-defective mutants suggesting that combining STAT3 and MTOR inhibitors (such as ABI-009) can be exploited as a novel therapeutic approach to treat drug-resistant GBM. Although the activation of PRKAA/AMPKα-ULK1 in the absence of STAT3 is not entirely unexpected, what is interesting is how these pro-autophagy pathways are inhibited in GBM. We hypothesize that the GBM microenvironment plays a crucial role in regulating these pathways. Because STAT3 activation contributes to the GBM immunosuppressive microenvironment, this raises the possibility that STAT3, in cooperation with the heterogenous cell population of the tumor microenvironment, suppresses PRKAA/AMPKα to influence the metabolic cues that support tumor growth.

PROM1/CD133 is a pro-autophagy protein in untransformed cells, and our data show that PROM1 expression increases in *STAT3*-KO GBM cells and cells expressing phosphorylation-defective STAT3 mutants. The relationship between STAT3-dependent inhibition of autophagy and PROM1 (cytosolic) expression is novel and may provide valuable insights into GBM tumorigenesis and help identify new molecular markers that are predictive of GBM tumor recurrence and chemoresistance. It is tempting to speculate that *STAT3*- and PROM1-driven autophagy signaling pathways play key roles in modulating GBM plasticity and heterogeneity, thereby facilitating GBM tumor progression.

The concept of intratumoral heterogeneity is supported by the observations that distinct stem-like clones from the same tumor demonstrate variable gene expression profiles. Our data support the notion of cellular and molecular heterogeneity in GBM. We identified differences between LN229 and MT330 GBM cell lines and observed variability in STAT3 transcriptional regulation of autophagy-related genes. While STAT3 inhibits *ULK1* gene expression, it increases expression of *SQSTM1* and *BNIP3* genes in MT330 cells. In contrast, STAT3 has no effect on transcription regulation of *SQSTM1* and *ULK1* genes but alters their protein levels suggesting transcription-independent but autophagy-dependent regulation of SQSTM1 and ULK1 in LN229 cells. In addition, we found that STAT3 phosphorylation sites have differential roles in suppressing autophagy. Expression of the Y705F mutant in *STAT3*-KO LN229 cells activates PRKAA/AMPKα, whereas expression of the S727A mutant has no effect. Similarly, this mutant has no effect on ULK1 activity suggesting that the Y705 mutant is the key regulator of both PRKAA/AMPKα and ULK1 signaling in LN229 cells. In MT330 cells, expression of both Y705F and S727A mutants in *STAT3*-KO cells activates PRKAA/AMPKα. The Y705F mutant increases ULK1 phosphorylation, but again the S727A mutant had no effect, showing that S727-STAT3 phosphorylation does not involve ULK1 to suppress autophagy in MT330 cells.

We also used *ULK1* siRNA and a pharmacological inhibitor of ULK1 and ULK2 (MRT68921) to demonstrate the importance of ULK1 activation in GBM autophagy. Inhibition of ULK1 by these strategies decreases autophagy and sensitizes *STAT3*-KO and phosphorylation defective mutant-expressing cells to apoptosis, demonstrating that concomitant inhibition of STAT3 and ULK1 promotes GBM cell death. Based on these observations, we propose dual targeting of STAT3 and ULK1 as a novel therapeutic approach for GBM treatment (Fig. 1). The combination of one or more small molecules capable of simultaneously targeting these two pathways may also be potentially effective in treating other cancers including GBM.

**Figure 1.**
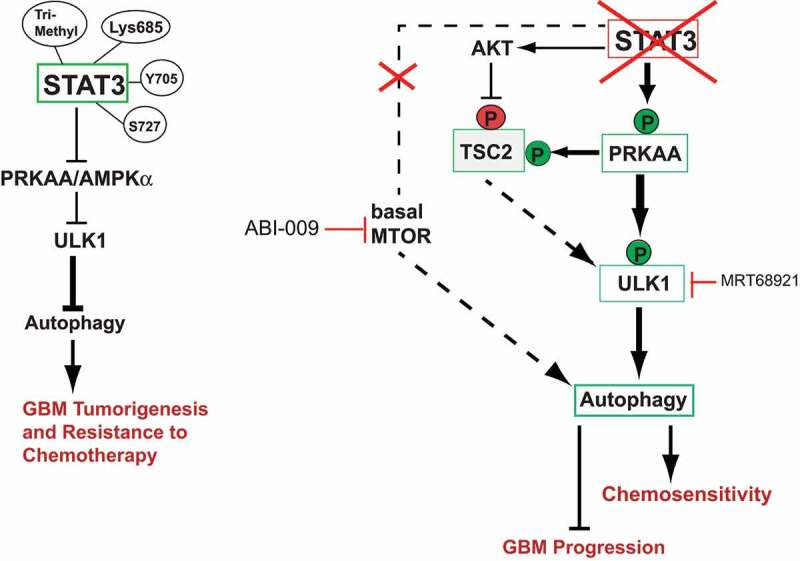
STAT3-dependent autophagy pathways regulating GBM tumorigenesis and chemoresistance. STAT3 PTMs inhibit PRKAA/AMPKα and ULK1, which suppress autophagy in GBM cells. This promotes GBM tumorigenesis and chemoresistance. *STAT3*-KO increases PRKAA/AMPKα activity, and *ULK1* gene expression and activity. These changes induce autophagy in GBM cells. Activated PRKAA/AMPKα in *STAT3*-KO cells phosphorylates TSC2 at S1387, which keeps MTOR activity in check leading to sustained autophagy. In addition, activated AKT in *STAT3*-KO cells inhibits TSC2 via phosphorylation at T1462, resulting in basal MTOR activity. This can inhibit ULK1, which is counteracted by ULK1 phosphorylation at S555, leading to autophagy. *STAT3*-KO does not alter basal MTOR activity, but MTOR inhibition by ABI-009, or ULK1 inhibition by MRT68921 in *STAT3*-KO cells can reduce GBM progression and increase chemosensitivity. →, Activation; ⊥, inhibition; (P) in red indicates phosphorylation causing inhibition; and (P) in green indicates phosphorylation causing activation.
